# A new electrospray method for targeted gene delivery

**DOI:** 10.1038/s41598-018-22280-2

**Published:** 2018-03-05

**Authors:** Stephan Boehringer, Paulius Ruzgys, Luca Tamò, Saulius Šatkauskas, Thomas Geiser, Amiq Gazdhar, David Hradetzky

**Affiliations:** 1 Institute for Medical and Analytical Technologies, School of Life Sciences, University of Applied Sciences and Arts Northwestern Switzerland, Muttenz, Switzerland; 2 Department of Pulmonary Medicine, University Hospital Bern, Bern, Switzerland; 3 Department of Biomedical Research, University of Bern, Bern, Switzerland; 4 Biophysical Research Group, Faculty of Natural Sciences, Vytautas Magnus University, Kaunas, Lithuania; 5 Graduate School for Cellular and Biomedical Science, University of Bern, Bern, Switzerland

## Abstract

A challenge for gene therapy is absence of safe and efficient local delivery of therapeutic genetic material. An efficient and reproducible physical method of electrospray for localized and targeted gene delivery is presented. Electrospray works on the principle of coulombs repulsion, under influence of electric field the liquid carrying genetic material is dispersed into micro droplets and is accelerated towards the targeted tissue, acting as a counter electrode. The accelerated droplets penetrate the targeted cells thus facilitating the transfer of genetic material into the cell. The work described here presents the principle of electrospray for gene delivery, the basic instrument design, and the various optimized parameters to enhance gene transfer *in vitro*. We estimate a transfection efficiency of up to 60% was achieved. We describe an efficient gene transfer method and a potential electrospray-mediated gene transfer mechanism.

## Introduction

The delivery of genetic material into a cell or tissue with the aim of altering the disease state is a highly promising approach. Various genetic materials such as DNA, RNA and mRNA have been tested for their treatment potential. The major hindrance to providing gene therapy for clinical applications is the lack of a simple, safe, effective and efficient method of delivering active therapeutic substances into the cell. The cell membrane acts as a powerful barrier to this aim and various approaches, based on viral and non-viral vectors, have been taken to try to achieve successful gene delivery inside the cell^1^. Although viral vectors tend to provide higher efficiency, several complications, including immune response, were identified^2^. On the other hand non-viral vectors based on lipids, peptides, polymers or nanoparticles offer a lower risk of inflammatory response^3^ but are inefficient^1^; the need for efficient novel vectors therefore remains^3^. Non-viral physical methods such as electroporation, sonoporation, magnetofection and hydroporation all have application limitations, either to specific diseases or organs^4,5^. These limitations of existing viral and non-viral gene delivery methods block the progress of gene therapy for clinical applications; innovative methods for gene delivery are thus required. In the current study we report and demonstrate a physical method to deliver genetic material into cells in an *in vitro* setting. Electrospray, also known as electro hydrodynamic atomization (EHDA), uses an electrical field to create and accelerate small sized droplets. This principle is used in different applications including mass spectrometry^6^, (thin) film deposition^7,8^, the fabrication of micro- and nanoparticles and their encapsulation^9^ and other related applications^10,11^. Electrospray for *in vitro* gene transfer was reported on cells pre-incubated with plasmid using water^12^, high conductivity liquids^13^, plasmid suspension^14^ and gold nanoparticles coated with plasmid^14^. All of these approaches describe a complex, hard to use technical set up consisting of a capillary, a mechanically adjusted working distance and a culture medium connected to a second counter electrode.

To make electrospray easy to use, two crucial points have to be considered: first, a well-defined working distance during application has to be maintained; second, the target has to be approached easily using a single device and a single access port, avoiding any additional interconnects or parts. Solving these problems will be a significant step on the path to easy-to-use targeted gene delivery.

In the current study, we demonstrate an electrospray device, defining its fabrication and design and report its *in vitro* application.

## Results

### Design and device set up

**Figure 1:**
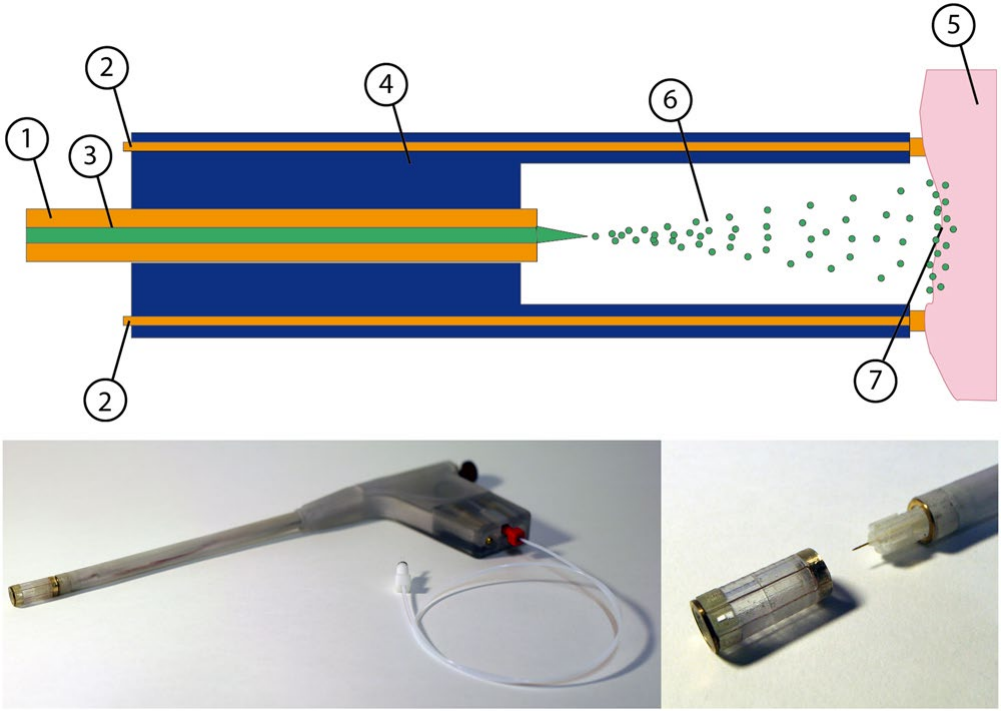
Schematic cross section of the electrospray device (**a**), consisting of the fluid delivery capillary (1) acting as primary electrode connected to high voltage, counter electrode (2) delivered media (3) single housing (4) target tissue (5) connected via the counter electrode (2) to ground. Functional prototype of the electrospray device (**b**) based on rapid prototyped rigid housing with an outer diameter of 10 mm. An electrical (not shown) and fluid interconnect are located on the handle. A close up (**c**) displays the working chamber (disassembled) with the integrated distal electrode and drug delivery capillary with the device. The outlet of the capillary is located in the centre of the working chamber when assembled.

The basic concept and design, shown in Fig. 1a, can be potentially applied within a lumen and be used for targeted gene delivery. It is based on a concentric arrangement of electrical conductive fluid delivering capillary (1) centred in a housing (4) and acting as primary electrode. The fluid (3) containing the plasmid to be delivered is dispersed into small droplets (6) by the electrical field established between the capillary and the targeted cells or tissue (5). Due to the target conductivity and the electrical interconnect within the instrument (2) the tissue acts as a counter electrode. The primary (1) and counter (2) electrodes are connected to a high voltage source, driving the electrical field to generate the electrospray. To provide a continuous delivery of fluid, the capillary is driven by a syringe pump. This basic concept is transferred into rigid device shown in Fig. 1b,c, to be used as a single port access instrument for *in vitro* or potential clinical applications.

**Table 1:** Mean value and standard deviation of droplet velocity of generated droplets of 370 mOsm solution at different electrospray voltages, grouped into sub groups at U_ES_ = 3 kV, d_wd_ = 4 mm, dV/dt = 20 µl/min, c_eGFP_ = 100 ug/ml.

Electrospray voltage U_ES_	group 1		group 2		group 3	
	<v_d_> [m/s]	<v_d_> [nl]	<v_d_> [m/s]	<v_d_> [nl]	<v_d_> [m/s]	<v_d_> [nl]
	V_g_ [nl] (V_%_ [%])		V_g_ [nl] (V_%_ [%])		V_g_ [nl] (V_%_ [%])	
2.7 kV	2.8 ± 1.3	1.1 ± 1.1	12.9 ± 3.9	0.2 ± 0.7	3.2 ± 4.4	9.9 ± 4.2
	611 (42%)		107 (7%)		724 (50%)	
3.0 kV	2.9 ± 1.3	1.4 ± 1.5	14.3 ± 3.5	0.2 ± 0.4		
	1040 (95%)		51 (5%)			
3.3 kV	3.1 ± 0.9	1.4 ± 1.5	15.5 ± 2.8	0.7 ± 0.8		
	833 (82%)		179 (18%)			

Focusing on the major group contributing to the volume delivered, the mean volume of droplets increases with increasing applied voltage, while the fraction of delivered volume reaches a maximum at an applied Voltage of U_ES_ = 3 kV.

The main application settings influencing the electrospray and thus expected to have an impact on the permeation of droplets into the cells are applied voltage (U_ES_) between primary and counter electrode, flow rate (dV/dt) of the fluid and working distance (d_wd_), the axial distance between the tip of the capillary and the counter electrode. The impact of these parameters on the transfection efficiency (η_T_) was investigated using eGFP reporter gene as an indicator for successful permeation of electrospray-delivered plasmid encoding eGFP (pMaxGFP) into the *in vitro* cultivated alveolar epithelial like cells (A549). The gene expression of eGFP was analysed 24 hours after electrospray treatment by fluorescence microscopy and flow cytometry. As the media delivered can affect the spray formation, the plasmid was dissolved (c_eGFP_ = 0.1 mg/ml) in hyperosmotic sucrose (c_Osm_ = 370 mOsm) solution. The generated spray was investigated using a high speed imaging system, generating a 2 second sequence containing 20,000 single frames to analyse the evolution of sprayed droplets. Image processing was used to derive the droplet volume (V_d_), and the velocity (v_d_) at impact depending on the parameters mentioned.

### General application and procedural settings

Initially a rough approach to applicable parameter range (d_wd_, U_ES_, dV/dt) was elaborated, focusing firstly on practical application criteria. In fact not all combinations of working distance and applied voltage are suitable, as increasing voltage at a given working distance or a reduction of working distance at fixed applied voltage may increase the risk of electrical discharge and should be avoided. Applicable potential for used working distance are U_ES_ = 3.2 kV @ d_wd_ = 3 mm; 3.3 kV @ 4 mm and 3.9 kV @ 6 mm. All applicable minimal working distance and maximum voltage combinations are shown in Supplement Table 1.

**Figure 2:**
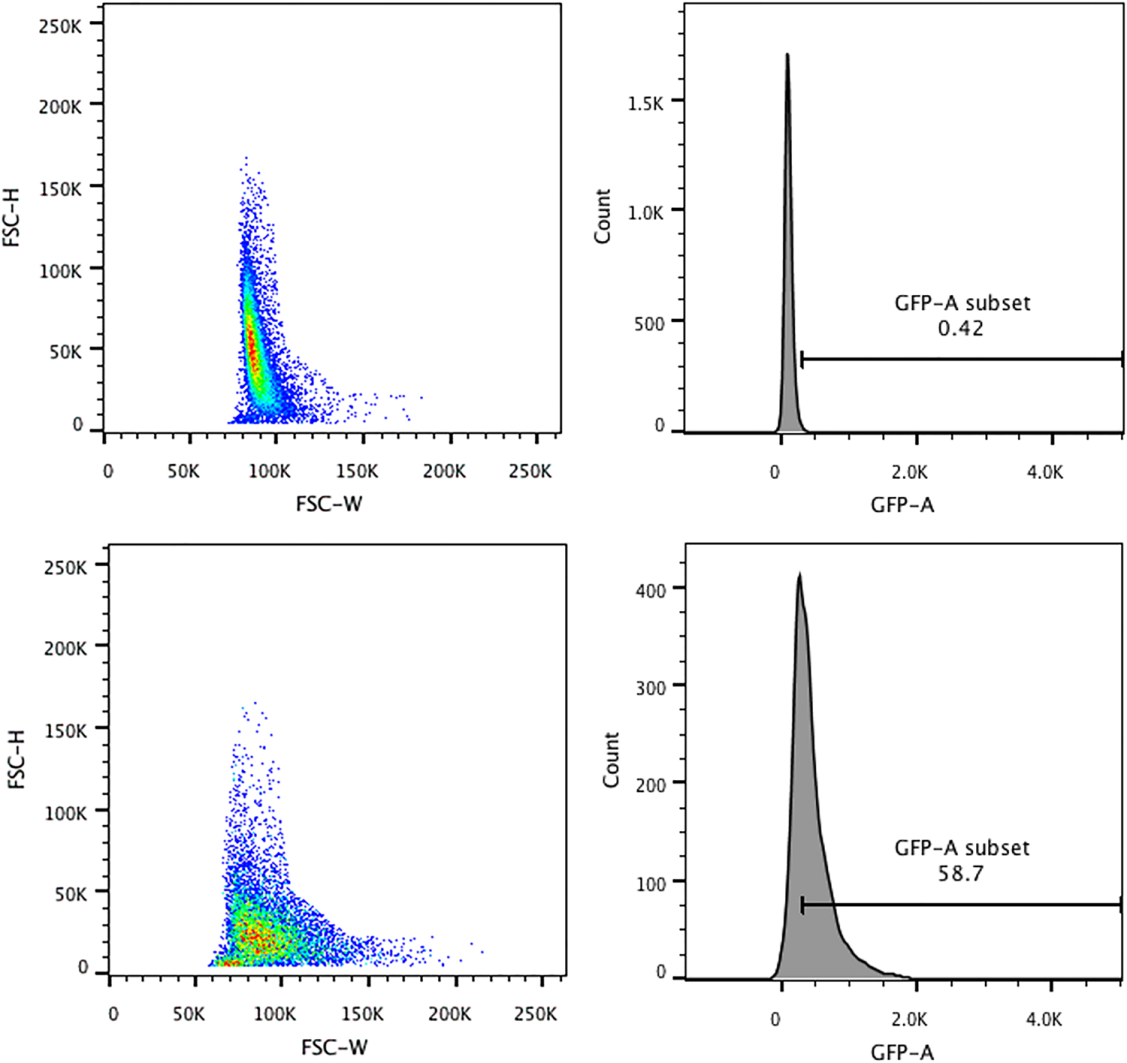
Flow cytometry to determine change in cell size after electrospray. After discarding cell debris, cell population with height and width forward scatter data and gating is shown for control cells (**a**,**b**) (no electrospray), and cells (**c**,**d**) electrosprayed with hyperosmotic 370 mOsm. During transfection cells were sprayed with sucrose based media with osmolarity of 370 mOsm. Working distance of 4 mm, flowrate of 20 µl/min with voltage of 3 kV was used for electrospray. Analysis was performed 24 hours after spraying.

Furthermore, the impact of flow rate on the cellular monolayer was identified. Using flow rates of dV/dt = 40 µl/min and above, electrospray treated cells detached from the plate. A flow rate of dV/dt = 20 µl/min, representing the lower limit of the system, did not cause damage or detachment of the cell monolayer and was used for further experiments. 24 hours after electrospray, flow cytometry height and width forward scattering measurements revealed altered cell size (Fig. 2). Electrosprayed cells (Fig. 2c) sucrose medium showed comparable cell size to control group (Fig. 2a).

**Figure 3:**
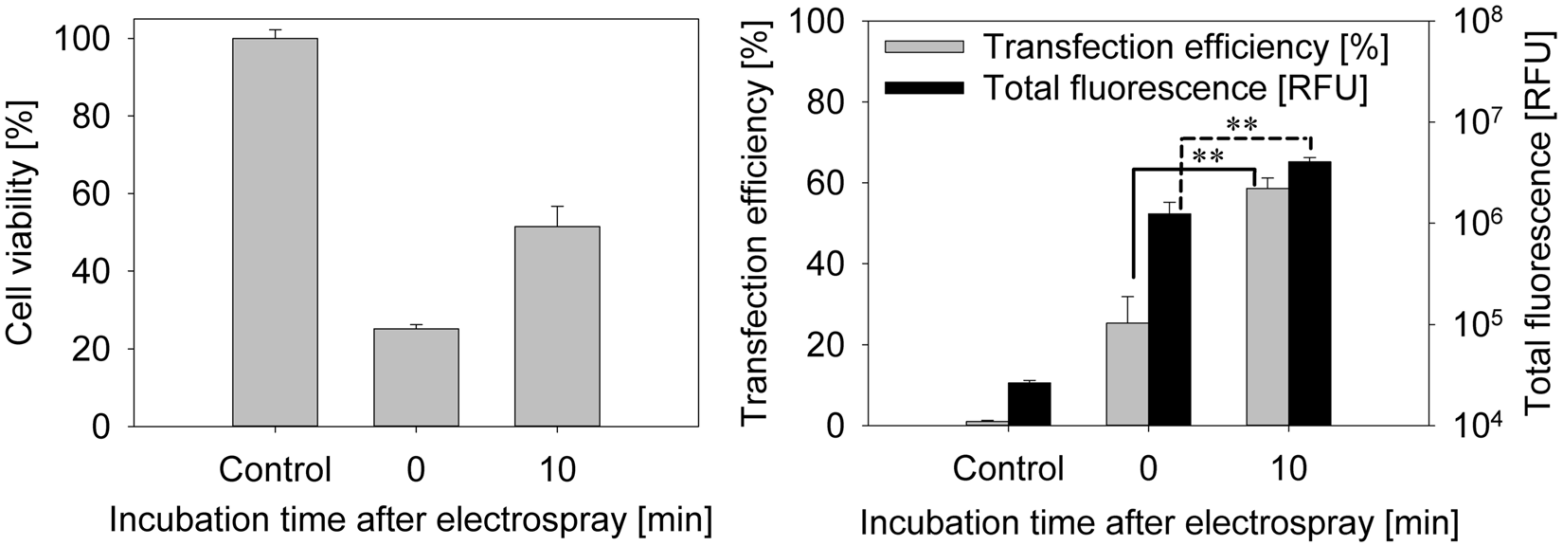
Incubation time after electrospray on cell viability was evaluated using 370 mOsm medium (**a**). Cell GFP plasmid transfection efficiency (**b**) and total fluorescence using electrospray is shown when t_inc_ = 0 min and 10 min incubation time after electrospray experiment was performed. Flow cytometer fluorescence measurements were chosen for cell transfection efficiency and total fluorescence representation. Cells were sprayed with sucrose-based media with osmolarity of 370 mOsm. Working distance of 4 mm, flow rate of 20 µl/min with voltage of 3 kV was used for electrospray. Analysis was performed 24 hours later.

**Figure 4:**
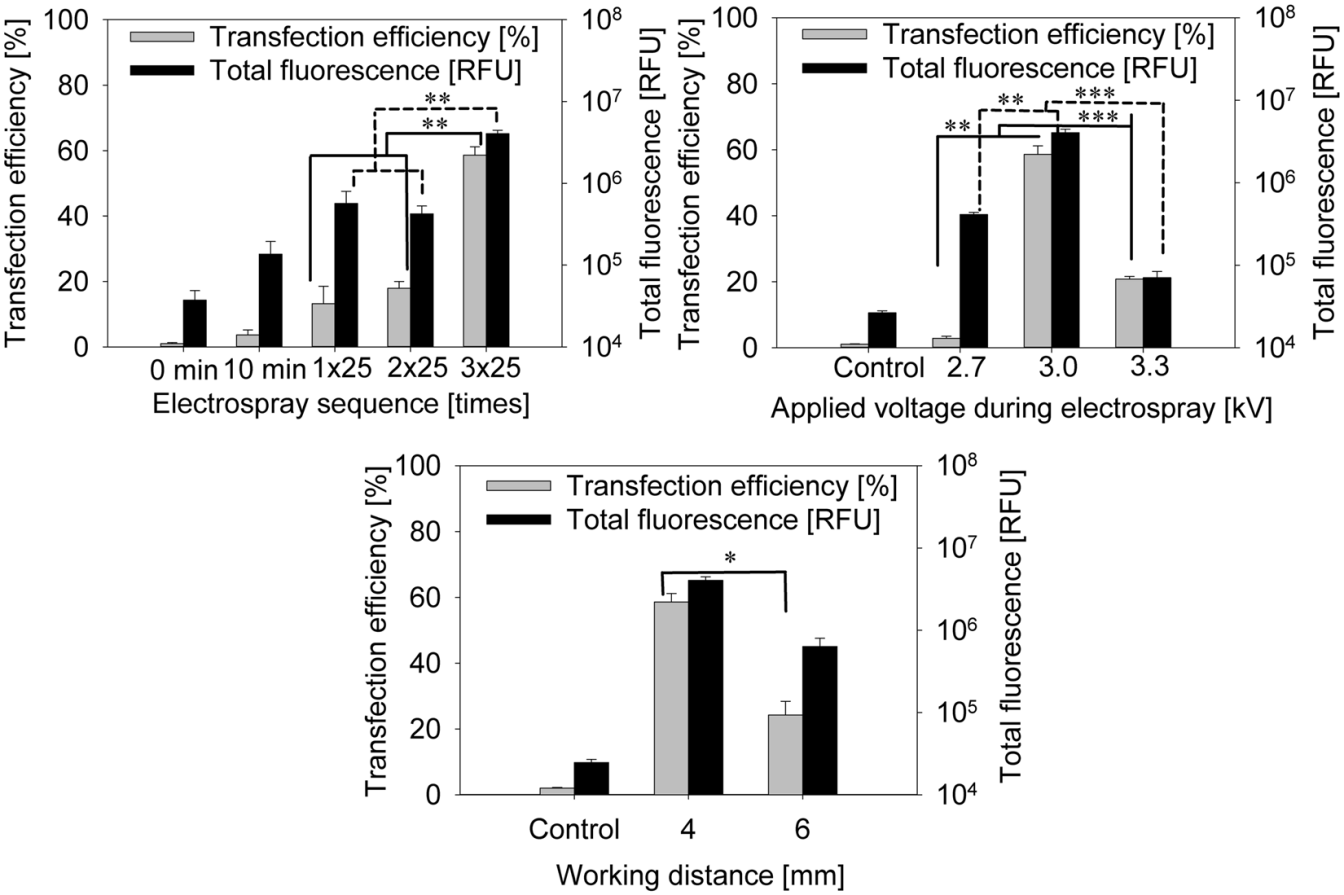
Cell GFP plasmid transfection efficiency and total fluorescence using electrospray in different sequences (**a**): 25 µl (1) 25 µl + 25 µl (2 × 25 µl) and 25 µl + 25 µl + 25 µl (3 × 25 µl). Flow cytometer fluorescence measurements were chosen for cell transfection efficiency and total fluorescence representation. Cells were sprayed with sucrose-based media with osmolarity of 370 mOsm. Working distance of 4 mm, flow rate of 20 µl/min with voltage of 3 kV was used for electrospray. Analysis was performed 24 hours later. Effect on transfection efficiency and total fluorescence of changing voltage (**b**) or working distance (**c**) with electrospray mediated pMaxGFP plasmid. Cells were sprayed with sucrose based media with osmolarity of 370 mOsm. Voltage range from 2.7 kV to 3.3 kV was used for electrospray (**b**), and working distance from 4 mm to 6 mm (**c**) was evaluated.

The electrospray mediated transfection efficiency using hyperosmotic sucrose based medium was elaborated. For reference, cells were incubated with GFP plasmid without electrospray. Fluorescence in the transfection efficiency gate was set to η_T_ = 1%. This limit differentiated cell autofluorescence and cells with GFP protein after GFP plasmid delivery. All presented experimental transfection efficiency percentage is calculated from cells that are adherent after 24 hours inside the area of electrospray device. The dead cells were gated out and were not included in analysis. In order to optimize the procedure, the incubation period following electrospray but before replenishing the nutrition media was modified. Different incubation times and their effect on transfection efficiency were investigated. Lag periods of 0 min, 10 min and 30 min after electrospray at room temperature were tested. After 10 min incubation time the transfection efficiency and total fluorescence increased to η_T_ = (58.6 ± 2.6)% and (4,048,403 ± 409,540) RFU respectively compared to η_T_ = (25.4 ± 6.5)% and (1,239,173 ± 372,707) RFU with no incubation (Fig. 3b). Moreover, 10 min incubation time also increased cell viability. As seen in Fig. 3a, 10 minutes’ incubation increased cell viability twofold, from (25.2 ± 1.1)% without incubation to (51.5 ± 5.3)%. However, an incubation period of 30 min led to major cell detachment (probably due to cell death). Therefore, transfection efficiency decreased to (3.0 ± 2.1)%. Delivering larger volumes (V > 25 µl) results in increased humidification of the inner walls of the electrospray device, thus increasing the risk of electrical discharge between the housing and the capillary. Although this electrical discharge is not expected to harm the targeted tissue, it should be avoided. Delivery volume of V = 30 µl was the upper limit of the electrospray device within a single application. In order to increase the delivered volume above this limit, a subsequent delivery sequence of V = 25 µl at dV/dt = 20 µl/min, resulting in a delivery time of t_d_ = 75 s (derived from V/(dV/dt)) per single application with an intermediate device cleaning and drying phase (t = 30 s) was elaborated and tested. After every 75 seconds’ spray the working chamber was cleaned for accumulated fluid. A time interval of 30 seconds was maintained between each spray as shown in Fig. 4a. Three different spray sequences were tested: single spray, two sprays or three sprays of 25 µl each for 75 s, with a time interval of 30 seconds between each spray.

Electrospray transfection results of these sequences are shown in Fig. 4b (Supplement Figure 1). Sequences of V = 25 µl and V = 25 µl + 25 µl electrospray gene deliveries were less effective than transfection efficiency deviated from η_T_ = (8.9 ± 2)% to (17.9 ± 2)%, whereas sequences of 25 µl + 25 µl + 25 µl show significantly higher transfection efficiency reaching (58.6 ± 2.6)%. The same tendency is seen when observing total fluorescence. Comparing the three sequences (a) 25 µl, (b) 25 µl + 25 µl and (c) 25 µl + 25 µl + 25 µl electrospray sequence total fluorescence increased by around 8 times when (c) 25 µl + 25 µl + 25 µl sequence was applied. The most efficient delivery was provided by applying a sequence of 3 single applications with two intermediate cleaning phases (Fig. 4).

Further, procedural and design parameters such as applied voltage (from U_ES_ = 2.7 kV to 3.3 kV) and working distance (from d_wd_ = 3 mm to 6 mm) were optimized. At working distances below d_wd_ = 3 mm, discharges were observed even below U_ES_ = 2.7 kV. Above d_wd_ = 6 mm and keeping U_ES_ = 2.7 kV, transfection efficiency was significantly lower (Fig. 4c, Supplement Table 2). Unreliable results were obtained when applying the working distance of d_wd_ = 3 mm, which caused electrical discharge between the capillary and the targeted cells, leading to decreased viability.

Transfection efficiency using working distance of 4 mm was η_T_ = (58.6 ± 2.6)% and (24.3 ± 4.2)% at 6 mm working distance. However, total fluorescence did not differ significantly. Therefore, d_wd_ = 4 mm was identified as the optimal working distance.

Optimization of applied voltage was performed by testing the voltage ranging from U_ES_ = 2.7 kV to 3.3 kV (Fig. 4b). Using 2.7 kV resulted in low transfection efficiency. Interestingly however, transfection efficiency at 3.3 kV was lower in comparison to 3 kV. Transfection efficiency, at U_ES_ = 2.7 kV (2.85 ± 0.81)%, 3 kV (58.6 ± 2.6)%, 3.3 kV, (20.8 ± 0.8)% was observed while keeping flow rate constant at dV/dt = 20 µl/min and working distance of d_wd_ = 4 mm (Fig. 4b). A similar pattern is seen in total fluorescence. Therefore, the optimal voltage for our prototype induced electrospray transfection efficiency is U_ES_ = 3 kV.

The essential outcome of the electrospray optimization for pMaxGFP plasmid transfection is the set of operating parameters U_ES_ = 3 kV, d_wd_ = 4 mm, dV/dt = 20 μl/min, t_inc_ = 10 min, using 370 mOsm solution and a spray sequence of 3 × 25 µl.

### Fluorescence microscopy

**Figure 5:**
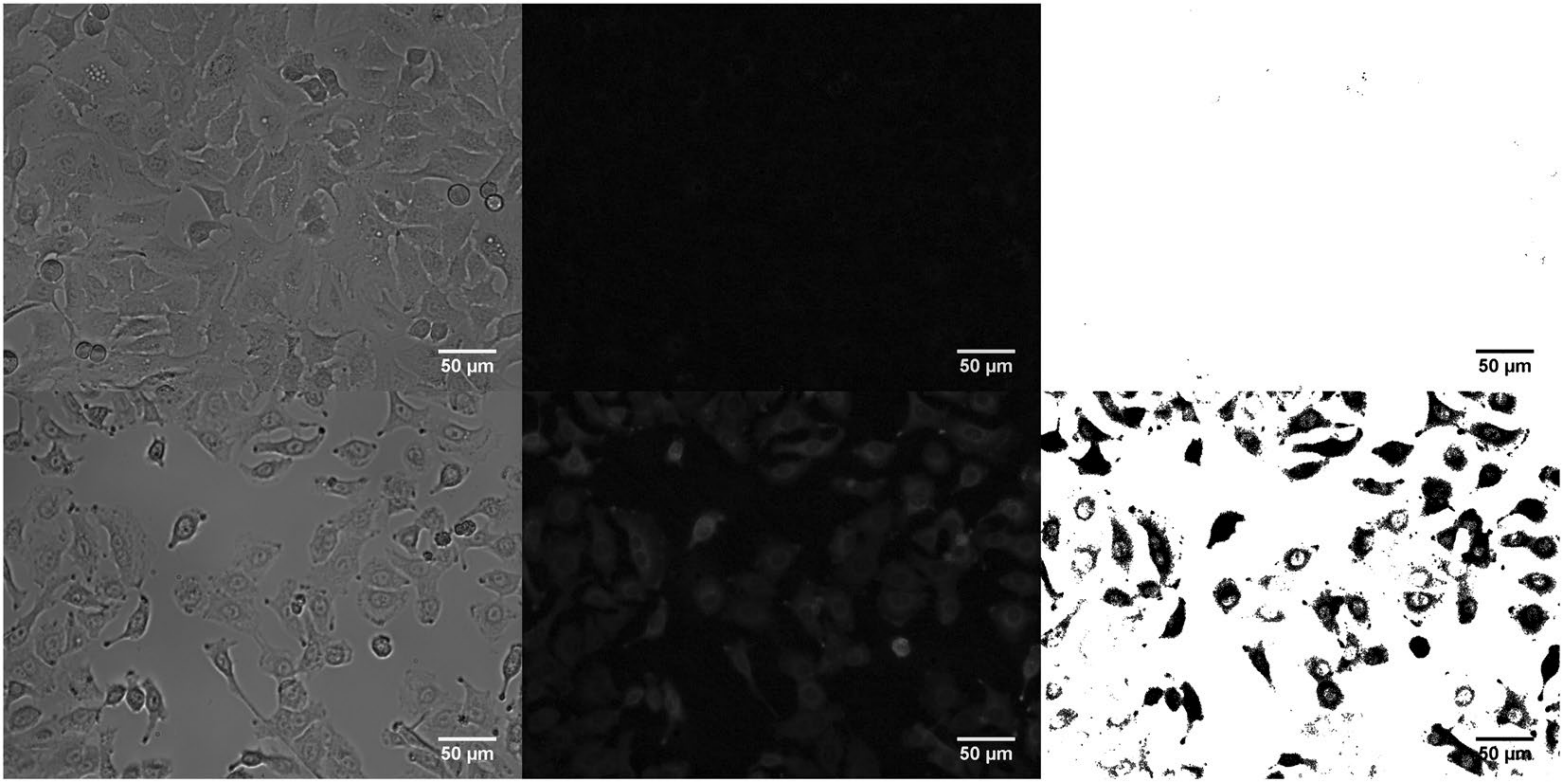
Fluorescent microscopy was performed for cell transfection visualisation. Cells with pMaxGFP and no electrospray (**a**–**c**). Electrospray mediated pMaxGFP transfection (**d**–**f**). Image processing was performed using Fiji image processing. Electrospray was performed with sucrose-based media with osmolarity of 370 mOsm. Working distance of 4 mm, flow rate of 20 µl/min with 3 kV voltage. 10 min incubation time was used. Analysis was performed after 24 hours.

In order to visualise the electrospray, mediated transfection cells were observed under a fluorescence microscope. Standardized procedural parameters were used (c_Osm_ = 370 mOsm, U_ES_ = 3 kV, d_wd_ = 4 mm, dV/dt = 20 µl/min, t_inc_ = 10 min) and electrospray mediated transfection with GFP plasmid was done for fluorescence microscopy imaging. Figure 5a–c demonstrate control figures where cells were treated with GFP plasmid without electrospray. Figure 5d–f show electrospray mediated transfection of GFP. Figure 5a,d are bright field images whereas Fig. 5b,e are fluorescent images. Figure 5c,f are processed images with Fiji software for better visualization, as mentioned in the methods section.

### Spray analysis

**Figure 6:**
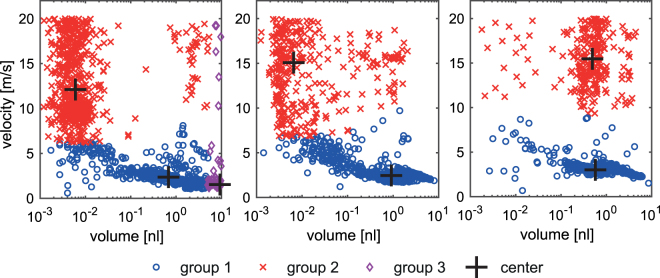
Volume dependent distribution of droplets with increasing applied voltage from U_ES_ = 2.7 kV (**a**), U_ES_ = 3.0 kV (**b**), and U_ES_ = 3.3 kV (**c**), when using eGFP in 370 mOsm sucrose media at a flow rate of dV/dt = 20 µl/min and a working distance of d_wd_ = 4 mm.

Following the trajectory of each single droplet of the evolving spray, the droplet velocity and cross sectional area were monitored, the latter to estimate droplet volume. Two different basic behaviours were observed; the droplets either (i) propagate as expelled from the capillary or (ii) split into smaller droplets. Droplets below V_D_ ≤ 4.2 pl or propagating with velocities larger than v_D_ ≥ 20 m/s were not suitable for reliable evaluation with the described high-speed imaging set-up. In general, the size dependent distribution of droplet velocity (Fig. 6) tends to group the droplets into two to three clusters. Each of these droplet groups is characterized by the centroid (indicated by the cross in Fig. 6) and represented by a mean estimated volume <V_d_> and velocity <v_d_>. Besides analysis of each individual droplet (v_d_, V_d_), the volume of each group (V_g_) and the fractional contribution (V_%_) of each group to the overall volume within the monitored image sequence were elaborated.

Different accelerating voltages were applied, within a range from U_ES_ = 2.7 kV to 3.3 kV, with hyperosmotic solutions using the standardized set of parameters. U_ES_ < 2.5 kV does not generate a reliable spray, while U_ES_ >3.3 kV results in a significant increase in the risk of electrical discharge. However, a higher voltage is expected to have a detrimental effect on the target and was therefore excluded.

The spray of hyperosmotic solution generally emerges in three groups at U_ES_ = 2.7 kV and two groups at U_ES_ = 3.0 kV and 3.3 kV (Fig. 6; Table 1). All sprays contain group of droplets with mean volumes slightly above <V_d_> > 1 nl (group 1) with comparable propagating velocities around 3 m/s, while the fractional contribution at 3 kV is maximum (V_%_ = 95%). A second group (group 2) emerges, with droplet volumes below ranging from <V_d_> = 0.2 nl to 0.7 nl, propagating at higher velocities (<v_d_> ≥ 12.9 m/s). This group contributes only a minor fraction to the delivered volume (V_%_ = 5% to 18%). An additional group (group 3) is observed at lower voltage (U_ES_ = 2.7 kV), where large droplets (9.9 nl) propagate with velocities comparable to group 1 and delivering about 50% of the fractional volume. As in group 1 the volume remains around 1 nl, an increasing the voltage has only a minor effect on the droplet volumes and speed of this group, but clearly affects the fractional delivered volume of this group and provides a maximum at 3 kV.

### Understanding electrospray mediated transfection

#### Gene transfer due to electrospray is not mediated by means of endocytosis

To have a clear understanding of how electrospray facilitates plasmid uptake into the cells, the process of endocytosis was evaluated. The idea that DNA delivery using electrospray can be associated with endocytosis comes from many electroporation and sonoporation studies^15–22^. Since electrospray, by its nature, is related with cell exposure to electric field (electroporation) or mechanical stimulation (sonoporation) we hypothetised that plasmid DNA uptake following electrospray could also be related with endocytosis.

For endocytosis inhibition Chloroquine diphosphate, Cytochalasin D, Amiloride, Filipin complex, and Hypertonic sucrose were used. Blocking of all known endocytosis pathways, however, did not have any effect on electrospray mediated gene transfer (Supplement Figure 3, Supplement Table 4).

#### Electrospray facilitates gene transfer by altering the cell membrane permeability

**Figure 7:**
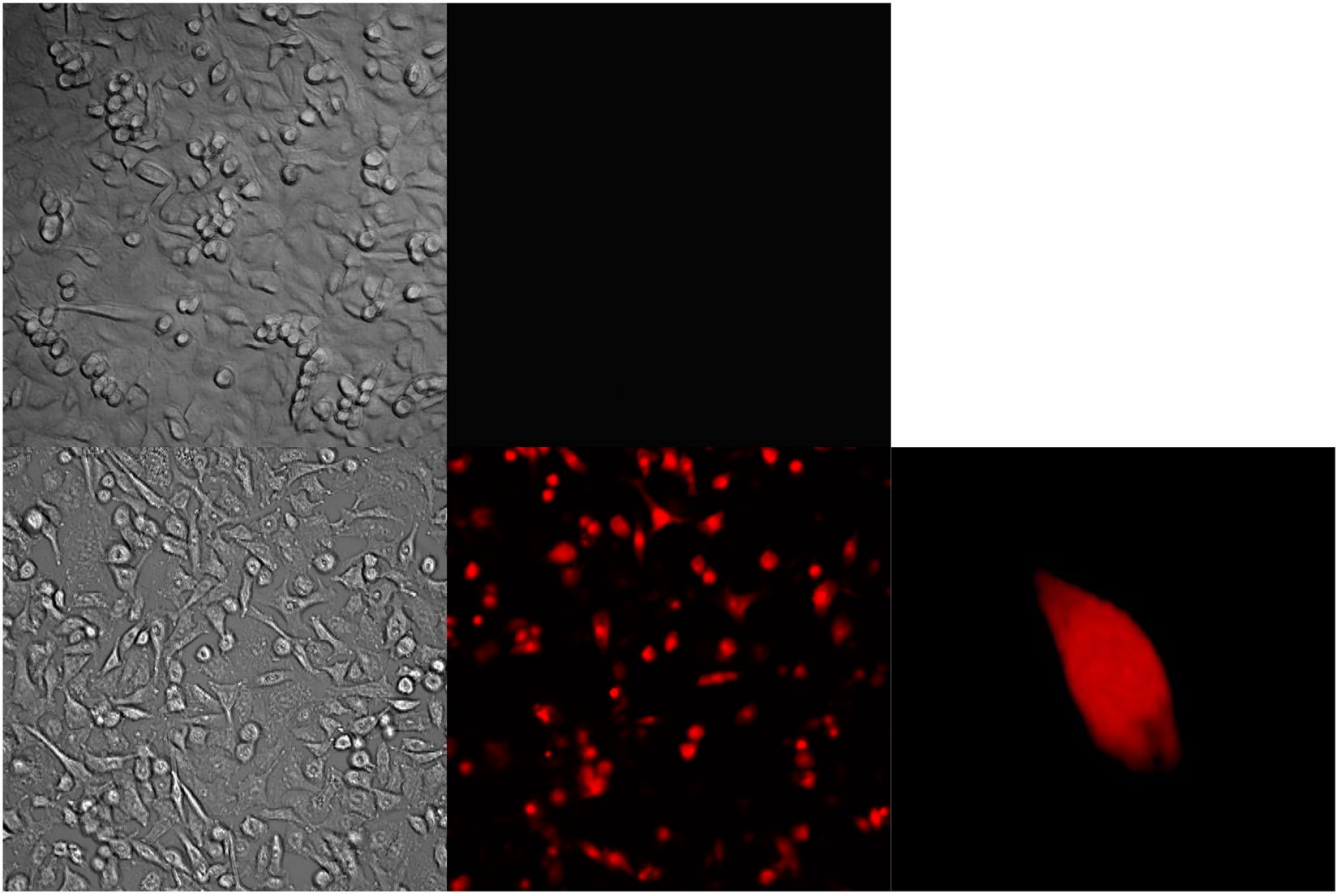
Electrospray with Propidium iodide (PI) was performed. Control cells only PI without electrospray (**a**,**b**), after PI electrospray (**c**,**d**), and 3D image using Fiji imaging after PI electrospray (**e**,**f**). Cells were sprayed with sucrose-based media with osmolarity of 370 mOsm. Working distance 4 mm, flow rate of 20 µl/min with voltage 3 kV was used for electrospray. 10 min incubation time was used.

To further understand a possible mechanism of electrospray mediated gene transfer, small molecule delivery to the cells during electrospray was examined. To check for the exogenous molecule transfer through cell membrane permeability, propidium iodide (PI) was electrosprayed (Fig. 7c–e). Cells exposed to PI but not treated by electrospray served as controls (Fig. 7a,b). Data showed that electrospray resulted in (72.79 ± 6.42)% PI positive cells. Moreover, with time there was an increase in PI inside the transferred cells.

PI transfer was evaluated using a confocal microscope for 10 minutes after the electrospray process. Results show statistically significant PI fluorescence changes when comparing before (2,892 ± 1,525) CTCF and after (16,233 ± 1,503) CTCF electrospray (Supplement Figure 2). Further, PI in-cell measurements reveal a statistically significant increase of PI fluorescence over time between 120 ss (16,233 ± 1,503) CTCF and 600 s (22,707 ± 1,928) CTCF. This indicates that PI molecules are still being transferred to the cell after the process of electrospray. These results correlate to Fig. 3, demonstrating that transfection efficiency changes when incubating cells for 10 minutes after electrospray.

#### Electrospray mediated gene transfer in subcutaneous mice tumor *in vivo*

**Figure 8:**
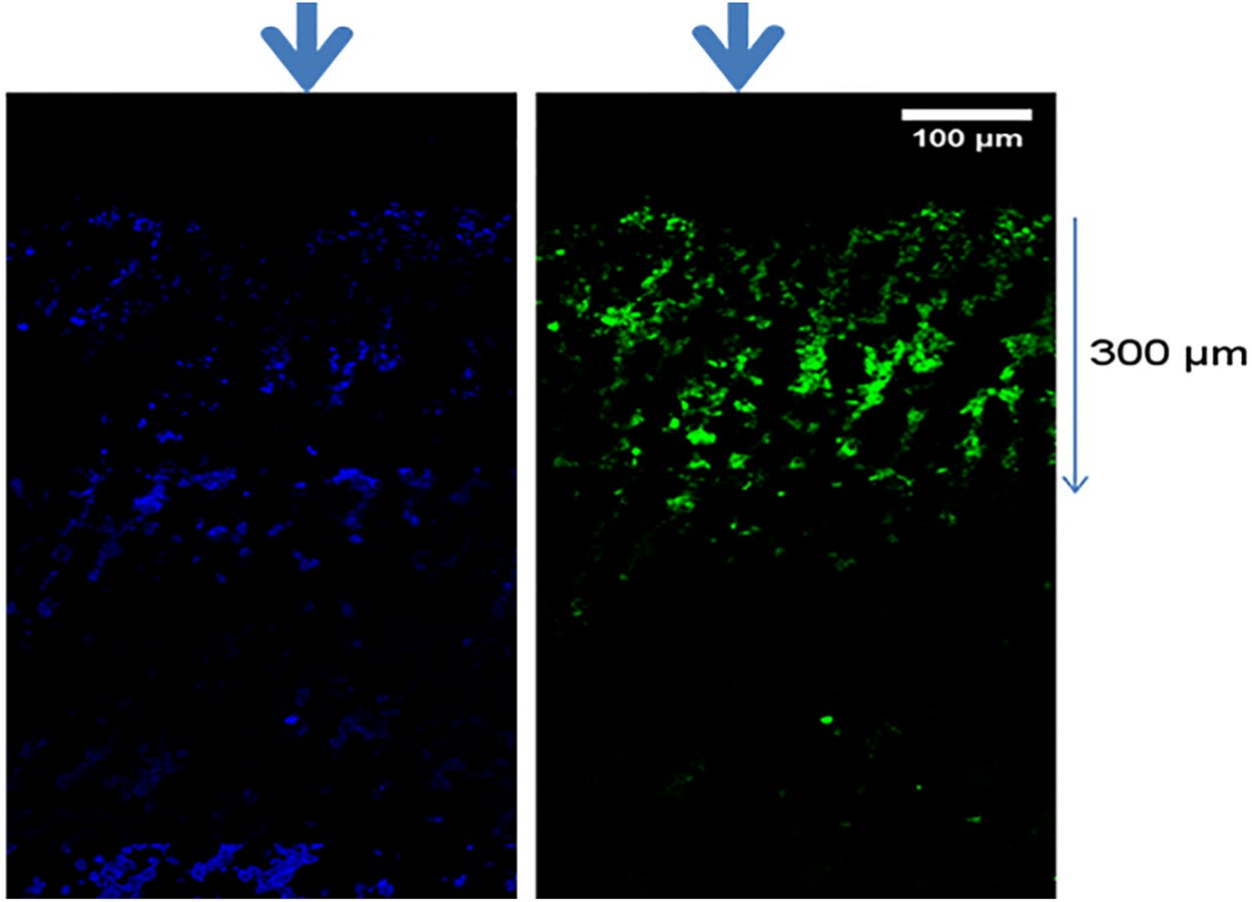
eGFP electrospray was performed on the subcutaneous mice tumor, and fluorescence microscopic images were taken 24 hours later. DAPI stain on the left and GFP stain on the right image. Arrow indicate the direction of electrospray.

Electrospray mediated gene transfer in the subcutaneous mice tumor lead to increased fluorescence as compared to control tumors. A longitudinal section of the tumor was analyzed under microscope to evaluate the depth of penetration after electrospray. Approximately 300 µm of GFP positive area was observed in the tumor after electrospray (Fig. 8).

## Discussion

In the current study we demonstrate electrospray mediated gene transfer *in vitro*. Electrospray mediated gene transfer is efficient, and reproducible. High gene transfection was observed with optimised parameters of applied voltage, working distance, spray sequence and the solvent used to dissolve the plasmid.

Currently, electroporation is the most common non-viral physical gene transfection method used for *in vitro* applications^23^. It has moderate efficiency when used for cells in suspension but is not optimal for cells growing in monolayer^24^. Lipid based transfection reagents used for cells growing in monolayer usually have a low efficiency^25^. In the current study we optimized and tested electrospray mediated gene transfer on cells growing in monolayer. Optimization of the various procedural parameters revealed the impact on droplet velocity distribution and transfection efficiency of applied voltage, working distance, flow rate, incubation time after electrospray and replenishment of media and solvent. By increasing the applied voltage from U_ES_ = 2.7 kV to 3 kV the transfection efficiency increased while the fractional volume of droplets (1 nl) increased from 42% to 95%; volume and velocity remained almost constant. Simultaneously, a decrease in the distribution of high velocity droplets and larger (9.9 nl) droplets at lower velocity was observed, indicating that these droplets contribute less to transfection than droplets within approximately 1 nl volume at 3 m/s. Similar results have already been described by Ikemoto^13^, spraying various media without plasmids DNA on a preincubated mixtures of cells and plasmid DNA. Moreover, increasing the applied voltage to U_ES_ = 3.3 kV decreased the efficiency (20.8%) and reduced the fractional contribution of low velocity large volume droplets (82%). As the transfection efficiency tends to follow the fractional contribution of the droplets within 1 nl volume propagating at 3 m/s, it can be assumed that this fraction contributes significantly to an efficient transfection. Comparing all results obtained, optimal efficiency is related to a high fraction of larger droplets that travel at mean velocities in the range of <v_d_> ≈ 3 m/s. Within this fraction the droplet volume of around <V_d_> ≈ 1 nl provides the most efficient gene transfection *in vitro*. However, there are limitations to the evaluation methods we applied. The high speed video sequence is limited to 2 seconds, representing only a fraction of the overall spray procedure; due to memory capacity and evaluation time restrictions, it does not seem feasible to expand this approach to the overall procedure length; the observable droplets below the volume <V_d_> ≈ 4.2 pl are close to the detection limit and cannot be evaluated in more detail; the estimation of droplet volume was based on the number of pixels covered by each droplet on the image; droplet generation based on modification of solvents, working distance and applied voltage does not provide independent adjustment of droplet volume, velocity and fraction. Finally, it has to be considered that the environmental conditions of the high speed imaging and cell culture experiments are not the same, and the selection of observed parameters may not be complete.

In summary the emerging spray mode can be categorized by droplet volume and velocity, and clustered as larger slower and smaller faster droplets. The larger slower droplet fraction contributes more to transfection efficiency than the smaller faster droplets. In particular, a volume around <V_d_> ≈ 1 nl seems to be the most suitable for gene transfection. These conditions may be obtained at an optimized technical and procedural parameter set. Applying a close working distance led to discharges and a distance of more than 4 mm did not yield efficient transfection. Similarly, at higher flow rates, the cells were detached and no transfection was observed; however, at a reduced flow rate electrospray mediated gene transfer was observed. Some essential aspects were reconsidered on observing very low transfection efficiency after cells were replenished with media immediately after electrospray. Therefore, an incubation period was introduced between electrospray and the addition of media to the cells, considerably increasing transfection efficiency. This observation led us to speculate that the electrospray mechanism might be related to alteration of the cell membrane permeability. Since large volume, slow moving droplets yield maximum transfection, endocytosis as a possible mechanism for gene transfer was speculated upon. Endocytosis is considered as a possible mechanism for gene transfer involving chemical based transfection methods based on cationic lipids and polymers^25^ and physical methods like electroporation and sonoporation^18,19^. Interestingly, in presence of endocytosis inhibitors, electrospray mediated gene transfer was observed, without any significant decrease in gene transfection. When measured with live cell imaging, fluorescence intensity initially increased after PI electrospray but later stabilized, indicating probable remodelling of the cell membrane^26^. We hypothesise that electrospray mediated stress on the cell membrane facilitates gene transfer; however after the stress was reduced, the cell membrane was gradually repaired. Moreover, we also observed eGFP positive cells in the mouse subcutaneous tumor indicating that this procedure can be potentially applicable *in vivo*. A detailed mechanistic study to understand the process of electrospray mediated gene transfer is further required to prove our theory. Electrospray is a very promising technology; however, the exact mechanisms of how it facilitates *in vitro* and *in vivo* gene transfer needs further clarification.

In conclusion, the electrospray method is a promising technique and provides an efficient method for gene transfection. Using the described approach, an optimized parameter set electrospray can be successfully used for *in vitro* gene transfer on adherent cells and on subcutaneous mice tumor. Moreover, with further technical development and improvement an instrument with single port access can be realized and used potentially for localized and targeted *in vivo* gene delivery, including intraluminal delivery. In the recent years essential modifications have been introduced using the minimal invasive techniques for therapeutic purposes especially for complex organ like lung^27,28^ we speculate that in the future electrospray could be in cooperated for localized therapy. With optimal design and development, electrospray could substantially advance the field of targeted and localized physical gene delivery methods, thus expediting the clinical application of gene therapy.

## Material and Methods

### Electrospray device, system and procedure

#### Electrospray system

The basic configuration, as shown in Fig. 1, was conceived to design a new electrospray device. The figure elaborates the liquid delivery port (1), acting as a primary electrode, the counter electrode (2) connected to targeted cell or tissue (5), separated by a working distance and mechanically protected from the surrounding environment by a housing (4) within a single applicator, the electrospray instrument. While mechanically maintaining a predefined working distance, this concept could provide a reproducible application scenario by design. The overall setup consists of a high voltage electrical supply and a pumping system controlled by a personal computer connected to the electrospray device. The high potential supply, connected to the fluidic delivery capillary (1), creates the emerging electrospray within the working chamber (6), accelerating the generated droplets towards the targeted region (7).

The hardware infrastructure consists of an electrical power supply module (ISEG HV- Power Supply DPR 60 155 24 5 EPU), providing output voltage up to 6 kV of reversible polarity. A syringe pump module (Hamilton PSD/4) with a 3/2 way valve was used to deliver liquid media, in combination with a glass syringe (250 µl). This combination enables the delivery of up to V = 250 µl media at flow rates from dV/dt = 10 µl/min up to 30 ml/min. The control of the modules via an I/O interface module (Keithley KUSB3100) was established through a LabView® coded application.

#### Electrospray device

The functional prototype of the electrospray device (Fig. 1b) was designed as a rigid single port instrument with an outer diameter of 10 mm, incorporating the fluidic and electrical interconnect. The device housing was fabricated with additive manufacturing technologies (Objet 260TM Connex) from bio-compatible, clear photopolymer (PolyJet MED610 from Stratasys), while the fluidic port was a stainless steel (316L) capillary (29G) with 140 µm (length: 50 mm) inner diameter, electro-polished, and electroplated with gold (Au). The fluidic port was placed concentrically within the tubular housing (Fig. 1c); it was connected to the high potential of the electric power supply (U_ES_) and axially relocatable, thus enabling adjustable working distance (d_wd_). The working chamber module was attached to the distal tip of the instrument with an integrated bayonet mount (Fig. 1c), providing the opportunity to modify the length, diameter and design easily, while enabling electrical connection to the targeted tissue. The working chamber offered an adjustable working distance from d_wd_ = 0 mm to 10 mm, with an inner diameter of 6 mm and incorporated the electrical interconnect and the counter electrode (Au electroplated 316L). Optimizing the distribution of the electrical field within the working chamber using Finite Element Method (COMSOL Multiphysics® 5.0) indicated the requirement for multiple electrical connections (6-wired) within the housing of the working chamber to assure a symmetrical field distribution.

#### Electrospray procedure

Using predefined protocols, subsequent steps were defined initially and maintained through the whole characterization procedure. The protocol turns on the electrical power to a predefined value first, and the syringe pump second, establishing the electrospray process. The flow rate is defined by the syringe volume and delivery speed. Reaching the volume to be delivered the syringe was turned off first, and after a delay of 10 seconds, the power source was turned off. This delay was required, as the fluid systems of the electrospray device feature a capacitive behaviour. During the electrospray process the current was monitored and limited to a maximum of 100 µA, in order to prevent electrical discharge. As the procedural parameters (flow rate, volume, and applied voltage) are stored, the system could be operated in an easy-to-use single button mode.

### *In vitro* electrospray mediated gene transfection

#### Cell culture

Alveolar epithelial like cells (A549) (ATCC, USA) were grown in RPMI medium (Gibco, USA) supplemented with foetal bovine serum (10%) (Gibco, USA) and Penicillin/Streptomycin (1%) (Gibco, USA). For electrospray experiments cells were grown in collagen coated 24 well plates, using purified bovine collagen solution (PureCol Biomatrix) at a concentration of 60 µg/ml. 1 × 10^5^ cells were plated in the centre of collagen coated wells, suspended in 10 µl of culture media. To facilitate attachment, cells were incubated for 30 minutes at 37 °C and 5% CO_2_ and then replenished with 500 µl of growth medium. This cell monolayer in the centre of the plate was covered by the electrodes during the electrospray procedure. Before electrospray experiments cells were checked under the microscope for even distribution in the centre of the well.

#### Plasmid

pMax plasmid encoding eGFP (pMaxGFP) (green fluorescent protein) (Lonza, Switzerland) was used. pMaxGFP plasmid amplification and purification was done using GigaprepEndoFree plasmid extraction kit (Qiagen, Germany) following the manufacturer’s protocol. The plasmids were diluted in TE buffer and used at an eGFP concentration of c_eGFP_ = 100 µg/ml.

#### Application of electrospray *in vitro*

For electrospray the plasmids hyperosmotic sucrose based media (c_Osm_ = 370 mOsm, 12.67 g per 100 ml) were used. Detailed composition and osmolarity of the electrospray media is presented in Supplement Table 3.

To optimize the electrospray parameters, the impact of the procedural parameters applied: voltage (U_ES_ = 2 kV…6 kV), (b) working distance (d_wd_ = 3 mm…10 mm), (c) Flow rate (dV/dt ≥ 20 µl/min) and delivered Volume (V = 25 µl…75 µl) on transfection efficiency were elaborated. Cells were electrosprayed according to predefined procedural parameters with eGFP plasmid. Before electrospray, eGFP plasmid was kept on ice and used at a concentration of c_eGFP_ = 100 µg/ml. Different volumes were delivered: 25 µl, 50 µl or 75 µl; accordingly the amount of eGFP plasmid varied: 2.5 µg, 5 µg or 7.5 µg. Plasmid solution was loaded to the electrospray device, the growth medium was removed and the electrode was placed with counter electrode in direct contact with the A549 cell monolayer within a well plate, and electrospray procedure was applied.

After electrospray, the medium was replenished at different time points, to test its effect on transfection efficiency. After electrospray the cells were kept at room temperature for 5 min, 10 min and 30 min and 500 µl of complete growth medium was added. Eventually cells were incubated in 37 °C, 5% CO_2_ for 24 hours before further analysis.

#### Endocytosis inhibitors

To understand the possible mechanism of gene transfer after electrospray, we tested the process of endocytosis. Different endocytosis inhibitors were used to block four different defined endocytosis processes^29,30^. Cells were incubated for 30 min with endocytosis inhibitors before experiment and electrospray mediated gene transfer was performed. All inhibitors with final concentrations and stated endocytosis blockage pathway are presented in Supplement Table 4.

### Analysis of electrospray mediated gene transfection *in vitro*

#### Microscopy and Flow cytometry

24 hours after pMaxGFP electrospray mediated delivery to cells, the analysis by flow cytometry using flow cytometer (BD bioscience LSR II (SORP upgrade) equipped with an argon laser emission of 488 nm was performed (BD bioscience, USA). A549 cells were trypsinized with 0.25% Trypsin EDTA (Gibco, USA) centrifuged and suspended in 150 µl of PBS as single cell suspension. Gating was performed as shown in Fig. 2b,d. Cell duplets were removed by gating cell population on forward scattering height and width plot. A primary gate based on physical parameters (forward and side light scatter, FSC and SSC, respectively) was set to exclude dead cells and/or debris. The background level was estimated by normal cells. Cell debris and cell duplets were subtracted from measured cell population to obtain GFP protein fluorescence in cells (Fig. 2b,d). Threshold for GFP fluorescence measurements in control cells was set to 1%. Cell viability percentage was evaluated by normalising cell uptake time using a fixed amount of cells as described^31^.

During flow cytometry analysis 1 × 10^4^ cells were analyzed. The exception was only when experiments were done using deionized water, 0.3% ethanol and 3% ethanol. In these cases fewer than 1 × 10^4^ cells were obtained 24 hours after electrospray. The analysis was performed using Flow Jo software (Flow Jo enterprise, USA).

#### Microscopy

24 hours after electrospray cells were washed with PBS and eGFP expression was measured using an inverted fluorescence microscope (Leica DMI4000 B, Germany). Image processing was performed with Fiji open source software (ImageJ, NCBI, USA).

#### Exogenous molecule transfer to cells

For cell exogenous molecule transfer to cells experiment imaging was done using confocal laser scanning microscope Zeiss LSM 710. Propidium iodide (Sigma) at the concentration of 250 µg/ml was used as a reporter molecule for the indication of exogenous molecule transfer to the cell. A 488 nm argon laser was employed for propidium iodide excitation.

1 × 10^5^ A549 cells were plated on collagen coated 35 mm glass petri dishes 24 hours in advance. Electrospray with propidium iodide was performed while the petri dish containing cells was mounted on a confocal microscope and the image acquisition was done 30 seconds after electrospray.

#### Image processing

For image processing Fiji open source imaging software (Image J, NCBI, USA) was used. All fluorescent microscopy images obtained were in 1392 × 1040 pixel format. Fluorescent image background was subtracted using rolling ball radius formula using 50 pixel value. Afterwards images were turned to RGB colours and background noise was eliminated where signal was less than 3 pixels area.

#### Spray mode analysis

The electrospray process generated small sized droplets that accelerated towards the targeted region. These droplets with variations in amount, size, speed and propagation direction, were defined as spray mode. To investigate the influence of procedural (composition of delivered media, applied voltage) and design parameters (working distance) the emerging spray mode was observed using a high speed imaging system^32^ (MotionPro Y3-S2). Through an observation window within the working chamber with an optics assembly from Navitar (C-Mount coupler 1–6218, 1.0× adjustable adapter telescoping 1–6218, 6.5X UltraZoom, 3 mm FF, Detent, Coax 1-60191D) at a frame rate of 10,000 Hz images corresponding to a field of view of 5.565 mm × 3.366 mm (image size: 992 × 600) were captured and analysed. This method enables the tracking of droplets covering a cross sectional area larger than 10 pixel (0.3 mm^2^) up to a propagation velocity (v_d_) of around 20 m/s.

The high speed video images were obtained using the standard protocol while applying the electrospray device on an indium thin oxide coated glass slide as target. The videos cover a sequence of 20,000 frames (2 s), with a delay of 30 seconds after switching the high power source on, and were evaluated within a predefined region of interest (ROI), covering 62.5% of the working distance starting 25% beyond the tip of the capillary and ending 12.5% ahead the target. Droplet trajectories were detected by analysing droplets in each frame within the ROI and comparing their positions with the droplets in the preceding frame. Droplets for evaluation were only considered when their first appearance was ahead of and the last appearance beyond the ROI. The dispersion of droplets into smaller droplets was also considered and all generated droplets were tracked. The velocity and trajectory of each droplet was obtained from a set of subsequent images at a fixed frame rate. The velocity of a droplet corresponds to the last two frames evaluated, and describes the droplet speed at impact. For each droplet the cross sectional area (number of pixels covered) and the diameter in direction of propagation was elaborated. In contrast to the velocity the volume is estimated at the first appearance (first frame) of the droplet where drops are usually close to the focal plane. As the emerging droplet shape was not restricted to circular or elliptical shapes, the volume of each droplet was estimated assuming a droplet with rotational symmetry around the direction of propagation with its major axis corresponding to the obtained diameter and the cross sectional area corresponding to the cross sectional area of the droplet. As the focal depth is limited only droplets remaining within the focal range of around 0.5 mm during their propagation were considered. For evaluation, three independent image sequences per analysed parameter set were acquired and analysed. The elaborated droplet data were then merged within one data set for clustering.

To distinguish between noise and a droplet the cut off size for detection was set to 10 pixels which is equivalent to a 4.2 pl spherical droplet (r = 10 μm) using a 5.6 μm/pixel resolution, or a 2.1 pl ellipsoid droplet (r_1_ = 20 μm, r_2_ = 5 μm). Because the blur of the pixels has a high influence on drops with a size of just a few pixels we defined 4.2 pl as the low limit of volume resolution.

For evaluation the droplets were clustered based on their size and velocity. The numbers of selected clusters are derived from the visual interpretation of the silhouette plots for numbers of clusters ranging from single cluster to 6. For clustering k-means clustering or Lloyd's algorithm^33^, implemented in Matlab® were used.

The spray mode analysis was applied at a set of standardized parameters (U_ES_ = 3.0 kV, d_wd_ = 4 mm, dV/dt = 20 µl/min), and on variation of single parameter: working distance, applied voltage, or delivered media. The evaluated video was based on a sequence starting 30 seconds after initiating the spray, in order to obtain steady-state spray conditions. Each setup was based on three subsequent repeated measurements, while the spray head was removed and cleaned in between.

### *In vivo* electrospray mediated gene transfer on mice subcutaneous tumor model

#### Animals

C57BL/6J adult male mice were obtained from Janvier labs (France). Animals were kept ad libidum. Experiments were performed in accordance with the standards of the European Convention of Animal Care. The study protocol was approved by the University of Bern Animal Study Committee.

#### Subcutaneous Tumor model

Lewis lung carcinoma (LLC) cells were grown in DMEM media (Life technologies, USA) in 10% FCS (Life technologies, USA). The animals were injected with 2 × 10^6^ Lewis lung carcinoma LLC cells suspended in 100 µl of 1X PBS subcutaneously on both the flanks. 15 days later tumor volume was measured using the calliper and experiments were performed as explained below.

#### Analgesia and Anaesthesia

Mice were injected with buprenorphine (0.1 mg/kg) subcutaneously 30 mins before surgery. For anaesthesia Midazolam (Dormicum) 5 mg/kg, Medetomidine (Domitor) 0.5 mg/kg, Fentanyl (Fentanyl-Janssen) 0.05 mg/kg were administered intraperitoneallly (0.05 ml for 20 g mice). For protection of eyes Bepanthen (5% Dexpanthenol) was applied to the eyes. After electrospray the mice were administered intraperitoneally with antagonist, Atipamazol (Revetor) 2.5 mg/kg, Naloxon (Narconti) 1.2 mg/kg and flumazenil (Anexata) 0.5 mg/kg (0.05 ml for 20 g mice).

#### *In vivo* electrospray

The tumor was exposed by skin incision and the electrode was placed on the tumor surface for electrospray. Tumors were pMax GFP was added on the surface served as control. For invivo electrospray following parameters were applied as follows: voltage 3 kV, with working distance at 4 mm, and flowrate of 100 µl/min. After electrospray the skin was sutured using VICRYL 4.0 (Ethicon, Inc. USA). Mice were injected with antagonist subcutaneously (sc) for reversal of anesthesia as described above.

#### Tumor collection and microscopy

The tumor was resected out and placed in optimum cutting temperature (O.C.T.) formulation of water-soluble glycols and resins Tissue-Tek® O.C.T. Compound, (Sakura® Finetek,USA) and was frozen at −20 °C till further processing. The tumor cryosections was cut of 8 µm thickness using the cryostate Leica CM 1850 (Leica Biosystems, Germany). The section was mounted with cover slip using the vectashield anti fade mounting media with DAPI (Vector Laboratory, USA) and the sections were observed under LSM Zeiss 710 Confocal microscope (Carl Zeiss, Germany). The image (Supplement Figure 4) were processed using the Imaris software 8.2 Bitplane Switzerland.

#### Statistics

Statistical analysis was performed using Sigmaplot12 (Systat Software Inc. USA). Three independent experiments of each experimental condition were performed. Data is represented as mean and standard error of mean (SEM) (mean ± SEM). For statistical significance, a t-test with two tailed and paired settings was made. Significance is represented as *p ≤ 0.05, **p ≤ 0.01, ***p ≤ 0.001, ***p ≤ 0.0001.

## Electronic supplementary material

Supplementary Data
