# Supplementary material for: A new electrospray method for targeted gene delivery
Source: Sci Rep. 2018 Mar 5;8:4031. doi: 10.1038/s41598-018-22280-2 (PMC5838090; doi:10.1038/s41598-018-22280-2)
Supplement: Supplementary file 1 — Supplementary Data [file 41598_2018_22280_MOESM1_ESM.docx]

# Supplementary Data

# A new electrospray method for targeted gene delivery

Stephan Boehringer*^1^, Paulius Ruzgys*^2,3,4^, Luca Tàmo^2,3,5^, Saulius Šatkauskas^4^, Thomas Geiser^2,3^, Amiq Gazdhar*^2,3^, David Hradetzky*^1^

1) Institute for Medical and Analytical Technologies, School of Life Sciences, University of Applied Sciences and Arts Northwestern Switzerland, Muttenz, Switzerland

2) Department of Pulmonary Medicine, University Hospital Bern, Bern, Switzerland

3) Department of Clinical Research, University of Bern, Bern Switzerland

4) Biophysical Research Group, Faculty of Natural Sciences, Vytautas Magnus University, Kaunas, Lithuania.

5) Graduate School for Cellular and Biomedical Science, University of Bern, Bern Switzerland

- *Equal contribution

**Corresponding Author**

**David Hradetzky**
University of Applied Sciences and Arts Northwestern Switzerland,
School of Life Sciences, Institute for Medical and Analytical Technologies
Gruendenstr. 40, 4132 Muttenz, Switzerland
+41 61 4674 307
[david.hradetzky@fhnw.ch](mailto:david.hradetzky@fhnw.ch)

**Amiq Gazdhar**
Department of Pulmonary Medicine,
University Hospital Bern
3010 Bern Switzerland
Tel: +41316327634
[amiq.gazdhar@dkf.unibe.ch](mailto:amiq.gazdhar@dkf.unibe.ch)

| 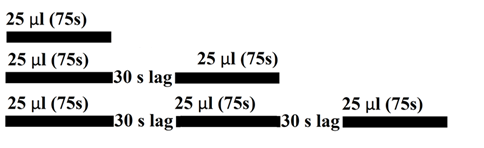  Figure 1: Electrospray sequences applied during the elaboration of general application settings. The delivery of V=25 µl per application represents the upper limit of the device. To increase the overall volume delivered a repetitive application with a cleaning phase in between was tested and optimized. |
| --- |

| 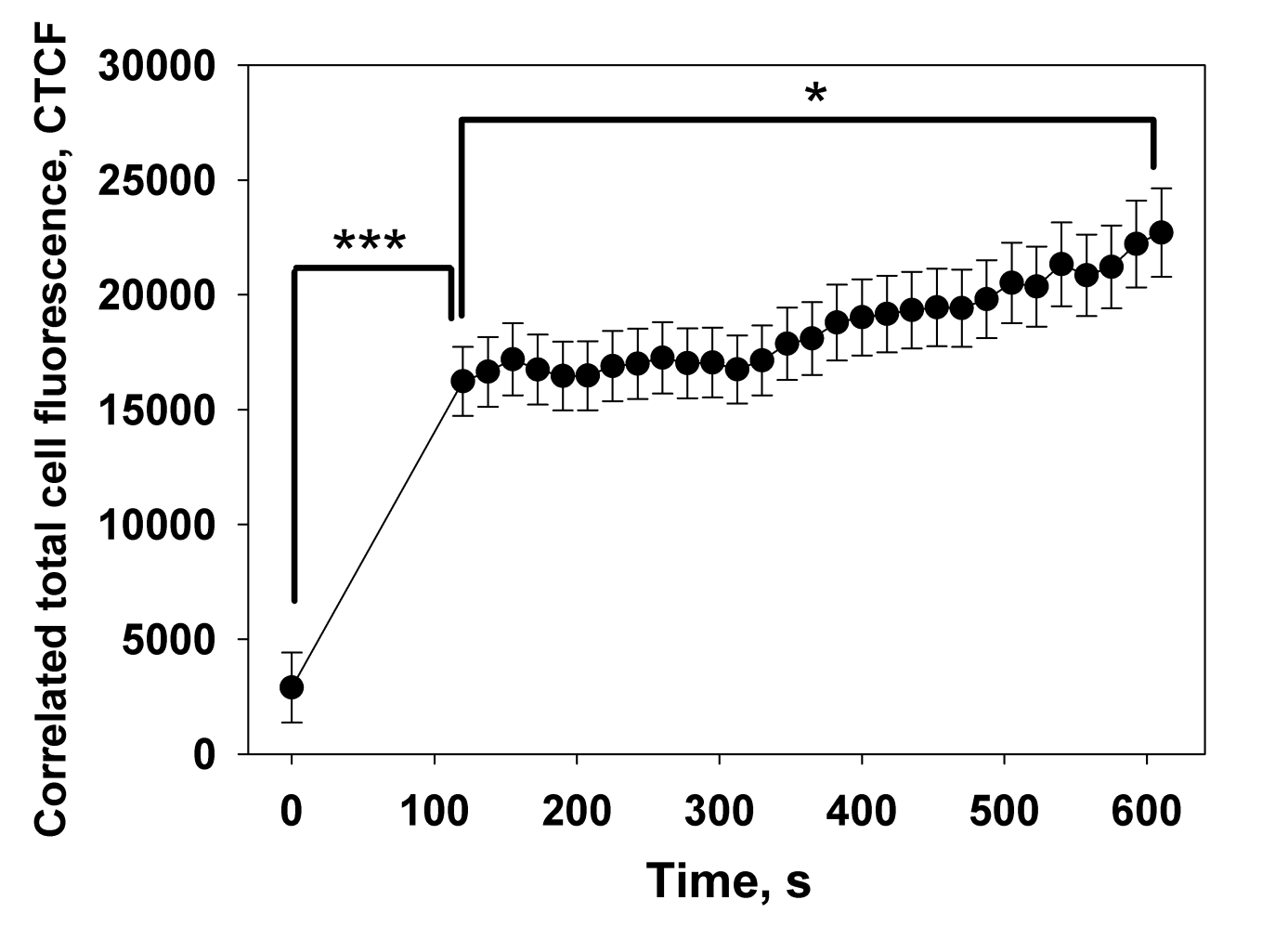  Figure 2: Time-lapse measured after electrospray with Propidium iodide (PI) on A549 cell line monolayer with confocal microscope. 0 seconds on timeframe is cell fluorescence in PI emission wavelength before electrospray. First time-lapse point after electrospray is 120 s and continued until 600 s (10 min). Cells were sprayed with PI solution in sucrose-based medium with osmolarity of 370 mOsm. Working distance 4 mm, flow rate of 20 µl/min with voltage 3 kV was used for electrospray. |
| --- |

| 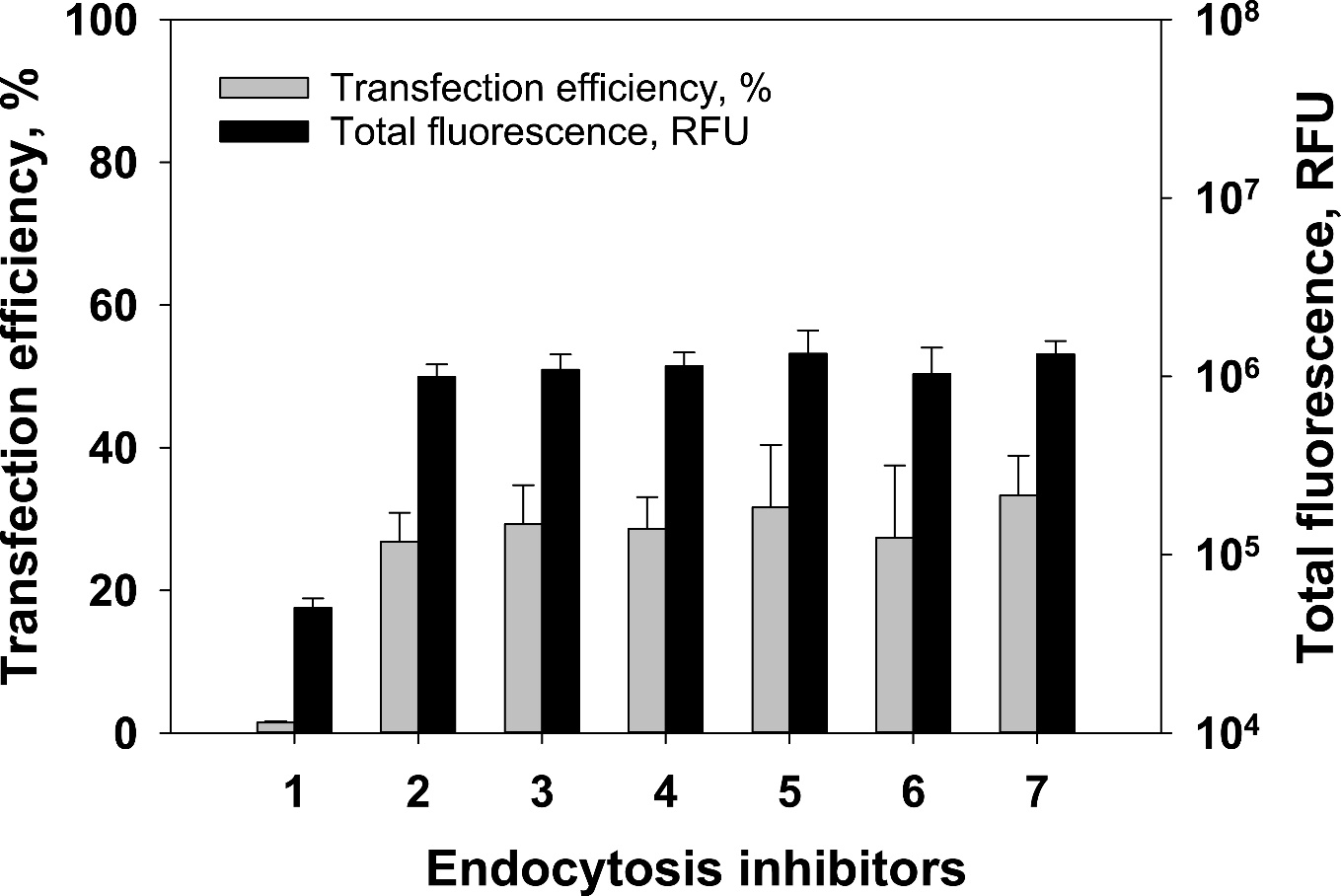  Figure 3: Electrospray mediated transfection efficiency after endocytosis inhibition. Cells were incubated with endocytosis inhibitors for 30 mins. Cells were sprayed with sucrose-based media with osmolarity of 370 mOsm. Working distance 4 mm, flow rate of 20 µl/min with voltage 3 kV was used for electrospray. 10 min incubation time was used. |
| --- |

Table 1: Combinations of working distance and maximum applicable voltage combinations without causing electrical discharge.

| Working distance (d_wd_) | 3 mm | 4 mm | 5 mm | 6 mm | 7 mm | 8 mm | 9 mm | 10 mm |
| --- | --- | --- | --- | --- | --- | --- | --- | --- |
| Max. Voltage (U_ES_) | 3.2 kV | 3.3 kV | 3.7 kV | 3.9 kV | 4.0 kV | 4.9 kV | 4.9 kV | 4.9 kV |

Table 2: Electrospray parameter range.

|  | Minimum values | Maximum values |
| --- | --- | --- |
| Working distance | 3 mm | 6 mm |
| Voltage | 2.7 kV | 3.3 kV |
| Flow rate | 20 µl/min | 20 µl/min |

Table 3: Composition of media tested for electrospray.

| Name | medium osmolarity | Sucrose (in 100 ml) | Ethanol |
| --- | --- | --- | --- |
| Hyperosmotic | 370 mOsm | 12.67 g | 0 ml |

Table 4: Endocytosis inhibitors used in electrospray experiments

| Endocytosis | Endocytosis inhibitors | Final concentration |
| --- | --- | --- |
| Clatrin mediated endocytosis | Chloroquine diphosphate, Hypertonic pressure | 200 µg/ml  450 mOsm (sucrose based) |
| Clatrin independent endocytosis | Filipin complex | 250 µg/ml |
| Macropinocytosis | Cytochalasin D | 2 µg/ml |
| Phagocytosis | Amiloride,  Cytochalasin D | 2 µg/ml  2 µg/ml |

| **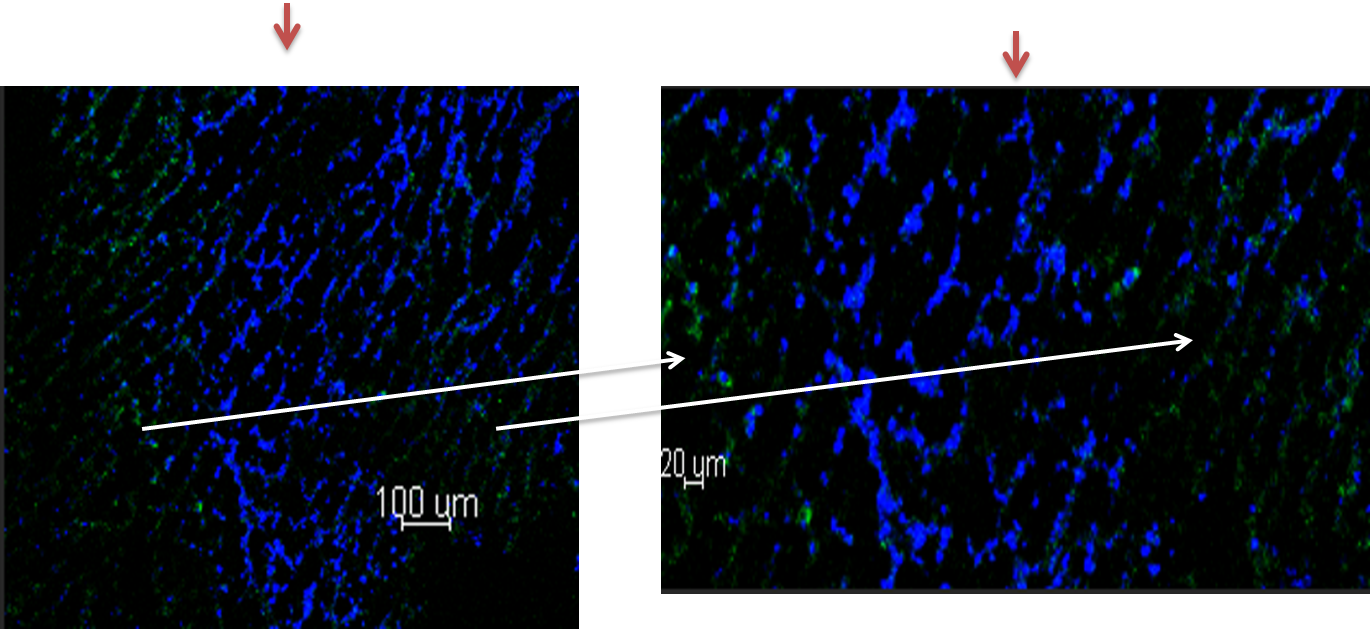**  Figure 4: eGFP electrospray was performed on the subcutaneous mice tumor, and fluorescence microscopic images were taken 24 hours later. The image on the left shows low maginification image, the image on the right is a digital zoom of the area transduced using Imaris software. Arrow indicate the direction of electrospray |
| --- |
